# Non-Contact Smart Sensing of Physical Activities during Quarantine Period Using SDR Technology

**DOI:** 10.3390/s22041348

**Published:** 2022-02-10

**Authors:** Muhammad Bilal Khan, Ali Mustafa, Mubashir Rehman, Najah Abed AbuAli, Chang Yuan, Xiaodong Yang, Fiaz Hussain Shah, Qammer H. Abbasi

**Affiliations:** 1 School of Electronic Engineering, Xidian University, Xi’an 710071, China; engrmbkhan1986@gmail.com (M.B.K.); roseyuanc@stu.xidian.edu.cn (C.Y.); fsgillani@stu.xidian.edu.cn (F.H.S.); 2 Department of Electrical and Computer Engineering, Attock Campus, COMSATS University Islamabad, Attock 43600, Pakistan; ali.mustafa@ciit-attock.edu.pk; 3 College of Information Technology, United Arab Emirates University (UAEU), Abu Dhabi 15551, United Arab Emirates; mubashir@uaeu.ac.ae (M.R.); najah@uaeu.ac.ae (N.A.A.); 4 School of Engineering, University of Glasgow, Glasgow G12 8QQ, UK; qammer.abbasi@glasgow.ac.uk

**Keywords:** COVID-19: smart sensing, OFDM, SDR, WCSI

## Abstract

The global pandemic of the coronavirus disease (COVID-19) is dramatically changing the lives of humans and results in limitation of activities, especially physical activities, which lead to various health issues such as cardiovascular, diabetes, and gout. Physical activities are often viewed as a double-edged sword. On the one hand, it offers enormous health benefits; on the other hand, it can cause irreparable damage to health. Falls during physical activities are a significant cause of fatal and non-fatal injuries. Therefore, continuous monitoring of physical activities is crucial during the quarantine period to detect falls. Even though wearable sensors can detect and recognize human physical activities, in a pandemic crisis, it is not a realistic approach. Smart sensing with the support of smartphones and other wireless devices in a non-contact manner is a promising solution for continuously monitoring physical activities and assisting patients suffering from serious health issues. In this research, a non-contact smart sensing through the walls (TTW) platform is developed to monitor human physical activities during the quarantine period using software-defined radio (SDR) technology. The developed platform is intelligent, flexible, portable, and has multi-functional capabilities. The received orthogonal frequency division multiplexing (OFDM) signals with fine-grained 64-subcarriers wireless channel state information (WCSI) are exploited for classifying different activities by applying machine learning algorithms. The fall activity is classified separately from standing, walking, running, and bending with an accuracy of 99.7% by using a fine tree algorithm. This preliminary smart sensing opens new research directions to detect COVID-19 symptoms and monitor non-communicable and communicable diseases.

## 1. Introduction

Countries around the globe have been experiencing a pandemic situation since December 2019. The outbreak of COVID-19 set up a concerning international public health crisis. As the outbreak continues to develop, the whole world is searching for possibilities to preclude the outbreak of the virus in new places, or to stop human-to-human interaction at places where the virus that originates COVID-19 was previously mingling. The public health departments in each county have taken the necessary steps to achieve these goals, such as implementing quarantine, which entails restricting human movement, maintaining a social distance from the rest of the public, or isolating healthy individuals who may not show any symptoms, with the goal of detecting virus-infected people early. Many states have legally permitted enforcement of quarantine from time to time when a new variant of COVID-19 starts spreading [1]. With the increasing number of health problems worldwide because of a lack of physical activity during the quarantine period, it is necessary to do indoor physical activities to prevent non-communicable diseases. They cannot spread from one person to another, but can last a long time. Cardiovascular diseases, cancer, diabetes, and other chronic respiratory diseases are categorized as non-communicable diseases. Sometimes, sudden falls due to physical activities may damage the human body, especially when a person is alone.

With the recent rapid technological advances, numerous monitoring systems such as camera-based, wearable, and ambient sensors-based technology are helpful for detecting falls to reduce fall-related injuries [2]. In this context, monitoring with portable sensors to detect immediate falls during physical activities not only helps in detecting falls but can also help with interventions before and after the fall [3]. The systems used to detect sports falls are based on the sports automatic recognition (SAR) system. They are designed to provide accurate measures and analysis in sports that have the potential to increase the efficiency and accuracy of exercises and increase health and safety. In common SAR systems, recognition can be attained through the machine and deep learning approaches by capturing data with inertial sensing and computer vision technologies [4]. Physical activities data measured by computer vision can be used for motion recognition and tracking. SAR systems include human detection and tracking, synchronization, and detection of targeted movements, depending on the type of sport and the camera settings [5]. SAR systems based on computer vision technology can provide coaches and athletes with prompt post-match analysis and real-time response before the next game. However, this system suffers from constrained environments, because the cameras are expensive devices and may not capture all subjects in the installed environment due to blind spots, affecting accurate measurement and performance analysis.

Another solution for detecting sports activities is inertial sensing technology, where the sensors are portable and consist of gyroscopes, accelerometers, and magnetometers. Wearable devices with onboard inertial sensors are commonly used in many applications such as rehabilitation, authentication and gait analysis, healthcare, human activities, diseases, navigation, etc. [6,7,8,9,10,11]. Recently, many researchers have developed and analyzed technologically advanced wearable inertial sensing-based sports activities monitoring systems for physical activities such as: running, jumping, cycling, golf, tennis, badminton, table tennis, football, baseball, basketball, and volleyball [12,13,14,15,16,17,18]. Although wearable sensing technologies are promising solutions for monitoring physical activities and detecting falls, they are not recommended in the pandemic situation because they may become a carrier for spreading the virus, and are uncomfortable for children and elders. Under these circumstances, non-contact sensing technology is a promising solution to control the spread of the virus, such as Wi-Fi, radar, and SDR-based human activities sensing technologies. These technologies are becoming popular in the modern era because they monitor human activities in a non-contact manner [19]. However, each technology has a trade-off between advantages and limitations, so when we talk about Wi-Fi-based sensing, it is low cost and easily accessible, but has portability and flexibility issues. On the other hand, radar-based sensing is generally used in military contexts, but a trade-off is the cost of equipment. SDR-based sensing provides an improved solution in terms of cost and performance. SDR-based sensing of human activities is cost-effective, portable, and flexible because software modification is possible without changing the hardware [20,21,22]. The main advantage of using SDR technology is that it can be exploited as Wi-Fi and radar technology as well. This initial research exploits the SDR technology-based non-contact smart sensing TTW by using artificial intelligence. The proposed system is a novel solution for monitoring falls during the quarantine period.

Following are the contributions of this research to monitoring physical activities during the quarantine period to prevent lifelong non-communicable diseases.

▪ Design of non-contact smart sensing system for monitoring falls by extracting fine-grained WCSI in the presence of walls.
▪ Enhancing the monitoring system by using portable, flexible, and multi-functional SDR technology.
▪ Intelligent monitoring accomplished by the use of machine learning algorithms. The optimal performance is evaluated by measuring classification accuracy, prediction speed, and training time for each algorithm.

The paper is structured as follows. In Section 2, related existing work on non-contact smart sensing for monitoring human activities by using Wi-Fi, radar, and SDR-based sensing technologies is provided for deeper insight. Section 3 provides an overview of the non-contact sensing platform used for development by exploiting the SDR technology. Section 4 is dedicated to the methodology used for extracting the WCSI data and building the classification model for physical activities. In Section 5, the accomplished results and their performance are presented. Lastly, Section 6 summarizes the performance of non-contact smart sensing using SDR technology, and future recommendations are suggested for improving the system.

## 2. Related Work

There are a lot of existing Wi-Fi technology-based platforms developed for human activities monitoring and detecting vital signs. The wireless sensor network (WSN) uses Wi-Fi technology to detect movements of the human body without a portable device in the operating area. Passive dynamic velocity moving people detection system (PADS) uses device-less detection to extract amplitude and phase information from the WCSI and exploit spatial diversity using MIMO systems; such a system uses commercial Wi-Fi devices to capture human body motions [23]. Human activity can be identified by the reflected Wi-Fi signals from the human body to create a unique pattern. A system that exploits CSI for detecting and monitoring human activities uses commercial Wi-Fi devices [24]. TTW human presence sensing systems use the Wi-Fi signals for moving and stationary people with a single Wi-Fi access point (AP). In this research, researchers have carried out the experiments in an empty room, in which a person moves or is stationary, and the channel frequency response (CFR) is analyzed for human activity [25]. Device-free-based solutions use Wi-Fi devices, which are generally available in homes and offices, to extract fine-grained CSI for analysis of human activities [26]. An untrained human vitality detection platform has been proposed that relies on basic Wi-Fi infrastructure to detect human movement in real-time. This system does not require any human effort to train offline or to calibrate manually. The platform can continuously monitor human activities for various purposes [27].

A wireless occupant activity recognition system (Wi-OAR) was developed for building management systems (BMS) to create user-friendly real-time environments for residents. The CSI extraction method based on Wi-Fi signals provides contactless user-centered services in offices to work intelligently. The fast and robust target component separation (FRTCS) algorithm is designed to evaluate both accuracy and time efficiency. This prototype was developed for different office environments with two commercial Wi-Fi devices [28]. A human activity recognition system used Wi-Fi signals to collect data from ten people doing sixteen different indoor activities. This system reduces costs and improves performance in various areas [29]. Wi-Motion used CSI data for extracting the phase and magnitude response to build the classification model for six diverse human activities [30]. A non-wearable and privacy-protective human activity detection platform used Wi-Fi signals for imminent smart buildings by extracting the images of wireless channel response [31]. The Wi-Fi-based system used deep learning algorithms with enhanced CSI features for human activity recognition [32].

Nowadays, Wi-Fi access points are very readily available everywhere, and the human presence between the access points provides a unique CSI. Machine learning is used to extract CSI data to classify human movements [33]. Wi-Fi technology is becoming increasingly popular in mobile sensing devices for monitoring daily human activities [34]. A non-contact sensing-based Wi-Run system uses commercial Wi-Fi devices to estimate human steps [35]. The wireless detection uses 5G C-band technology to record falls and body movements of people with high precision [36]. Passive Wi-Fi sensing monitors health conditions including breathing rate and falls [37]. A passive Wi-Fi system detects two-dimensional phase information for monitoring human falls [38]. Breathing and heart rate patterns are important indicators of a person’s physical health. A commercial Wi-Fi-based system was designed to analyze the changes in breathing and heart rate patterns. This system is inexpensive and convenient for continuous monitoring of health conditions [39]. Wi-Fall is a real-time system used to monitor the sudden falls of persons living alone, especially in old age. This system detects the fall of the human in a non-contact manner using a commodity 802.11n network interface card (NIC). This system achieves high accuracy for the fall detection of a single person [40]. RT-Fall, a non-contact sensing system, used commodity Wi-Fi devices for fall detection. This system is inexpensive for monitoring daily activities without attaching any device to the human body [41]. The Res-Beat, a non-contact sensing system, used commodity Wi-Fi devices for monitoring real-time respiration rate. The system analyzed bimodal WCSI data for breathing abnormality information by detecting peaks to evaluate respiration rates [42]. Wi-Fi technology-based sensing has the advantage of being easily accessible and low cost, but having limitations of portability and flexibility due to the limited number of OFDM subcarriers and fixed standards.

Radar technology is also used to monitor human activities and detect vital signs in the existing literature. A system based on ambient radar has been proposed to detect human activity in an indoor environment. The 7.8 GHz operating frequency detected human activity by sending 16 pulses per second. This system can differentiate between human movements to recognize different activities [43]. The Bumble-Bee radar-based system can efficiently capture micro-Doppler signatures for human movements for recognition in indoor environments [44]. A wireless sensing approach used a passive-Doppler radar to detect human body movement’s variations, recognizing abnormal respiration rate and various human physical activities to observe health condition. The wireless signals are used to detect human activity [45]. The radar technology detects large-scale body motions to improve the home life of older adults. This system classifies falls using radar spectrogram image data [46]. Radar technology is a promising solution, but has the potential risk of explosion due to released heat and is not used widely due to expensive hardware setup. Furthermore, the technology needs a line-of-sight (LOS) environment, i.e., no obstruction is recommended between radar and human, which limits the system’s physical deployment.

Recently, SDR-based sensing technology has been used to detect human activities and vital signs using wireless signals. A device-free system using smart sensing recognizes different human activities by extracting WCSI in an indoor environment. Human body motions were detected in a real-time setting using SDR equipment [47]. Blueprints of WCSI present distinctive variations caused by body motions, characterizing small and large-scale motions. SDR-based sensing technology exploits radio wave signals to extract human body motion patterns [48]. The SDR sensing-based platform used a deep learning algorithm-based convolutional neural networks (CNN) model to detect ankle movements [49]. The SDR sensing-based, non-contact identification platform classifies weightlifting activities performed by humans [50]. SDR technology is portable, flexible, scalable, and has multifunction capabilities [19,22]. The existing literature can be helpful in developing a COVID-19 platform to monitor human body motion, resulting in the diagnosis of various health issues, and monitoring of human activities in a non-contact manner. A summary of classification performance of monitoring health and vital signs by using non-contact sensing technology Wi-Fi, Radar, and SDR is given in Table 1. Although Wi-Fi, Radar, and SDR technologies are viable solutions for monitoring physical activities during the quarantine period, there are still limitations. In this research, we exploit SDR technology to overcome the limitations of Wi-Fi and Radar technology. The cost of SDR technology is low because it can be redefined through modification of software without changing or adding a new hardware setup. It is flexible because it can adopt any wireless standard by redefining software. It is portable because of the self-generating abilities of radio signals, and has multiple functional capabilities that can be exploited such as Wi-Fi, Radar, GSM, FM radio, etc.

## 3. Platform

**Figure 1:**
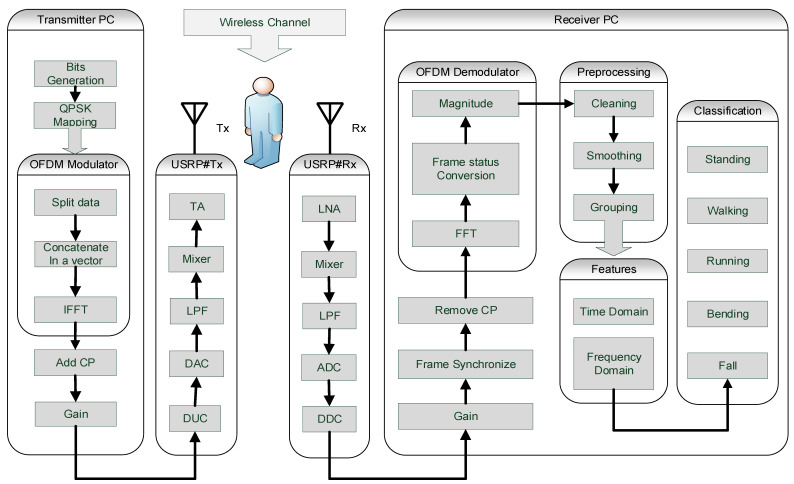
Non-contact smart sensing system overview.

The platform contains computers, SDR devices, and omni-directional antennas. The computers used for experiments are Lenovo, Intel(R) Core (TM) i5-7500 3.40 GHz processor, 12 GB RAM and Windows 10 64-bit operating system. The SDR devices used for experiments are universal software radio peripheral (USRP) B210, and the software is MATLAB Simulink version R2019a. The main functional blocks for the platform’s development are transmitter PC, transmitter USRP device, wireless channel, receiver USRP device, and receiver PC, as shown in Figure 1.

### 3.1. Transmitter PC

In transmitter PC operation, software-defined functionality is utilized to transmit a flexible OFDM frame. Initially, a signal of random bits is generated continuously at uninterrupted sample times and gets one channel per column. Data columns are buffered into frames by stipulating samples per frame. This input data signal used quaternary phase-shift keying (QPSK) digital modulation. Further, vector data was split into smaller subcarriers, and data of identical types concatenated to create contiguous output data. The inverse fast Fourier transform (IFFT) of all subcarriers is computed to transform frequency domain data into the time domain data with orthogonality between the subcarriers. A cyclic prefix (CP) is added to each data frame for avoiding inter-symbol interference (ISI). The adoptive gain is added to improve the strength of the transmitted signal. The software-defined hardware configuration block of USRP is used for flexible parameters modification, which is also the operation of the transmitter PC. The software-defined parameters are given in Table 2. These adjustable parameters can be redefined at any stage to improve the platform’s performance.

### 3.2. Transmitter USRP Device

The transmitter USRP device functions are digital up-conversion (DUC), digital to analog conversion (DAC), low-pass filtering (LPF), mixer, and transmit amplification (TA). These functions of the hardware device are fixed and cannot be altered.

### 3.3. Wireless Channel

**Figure 2:**
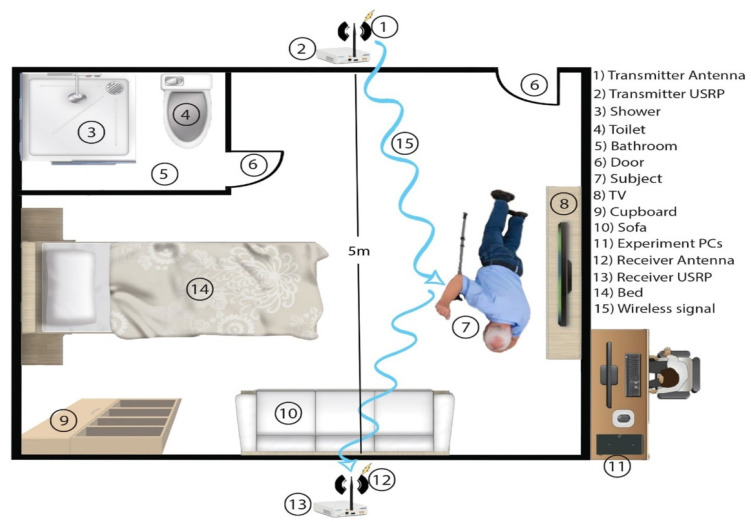
Experimental setup for collecting WCSI data using SDR technology.

The wireless channel is a room environment to collect the human movements of standing, walking, running, bending, and falling activities, as shown in Figure 2. The WCSI signal is collected through multipath due to the reflection of the human body in-between the two omni-directional antennas.

### 3.4. Receiver USRP Device

The receiver USRP device functions are low noise amplification (LNA), mixer, LPF, analog to digital converter (ADC), and digital down converter (DDC).

### 3.5. Receiver PC

In the receiver PC, the USRP hardware flexible receiver configuration block is used to modify and control hardware. The frame synchronization process is used to detect when the frame begins and helps remove the CP correctly. The FFT is applied to transform the time domain data into the frequency domain data. The frame status conversion sets the sampling mode of the output data frame. The amplitude response of the data is extracted to analyze the WCSI data in the frequency domain. WCSI data is in the raw form, which is further preprocessed by cleaning, smoothing, and grouping. Additionally, features are extracted to transform the WCSI data for meaningful analysis. Finally, three machine learning algorithms are applied to classify falls separately from standing, walking, running, and bending.

## 4. Methodology

The various steps involved in developing a physical activity monitoring and fall detection system by exploiting SDR technology and machine learning algorithms are discussed as follows:

### 4.1. Subject and Activities

In developing a physical activities monitoring system, we considered five healthy subjects performing multiple activities. The information about the subjects conducting the activities is given in Table 3. We considered standing, walking, running, bending, and fall activities for monitoring. Each subject performs each activity ten times.

### 4.2. Activities Data Collection

The activity data is collected in a small room, and the virtual experimental setup is shown in Figure 2. The distance between the antennas is 5 m and the subject performs an activity at the center position by moving his body. In standing, the position of the subject remains standing still; in walking, subject moves his legs slowly; in running, subject moves his legs quickly; in bending, subject bends his body; and in fall, the subject falls on the floor from a standing position. The reflection from the human body while doing different activities is collected as WCSI data.

### 4.3. Activities Data Extraction

The OFDM is used to extract fine-grained WCSI data at the receiver. The amplitude-based frequency response for each activity will be collected for 10 s. The information includes subcarriers, OFDM frames, and the time taken to perform the activity. Time and frames can be articulated as the number of frames received in a unit of time. The data sampling time is set based on the device sample rate by varying interpolation and decimation values at the transmitter and receiver, respectively. Each experiment frequency response H(jw) of WCSI data is expressed in Equation (1):

(1) $$H{\left(j\omega \right)}_{Experiment}=\left[\begin{array}{cccc} H{\left(j\omega \right)}_{11} & H{\left(j\omega \right)}_{12} & \ldots & H{\left(j\omega \right)}_{1s}\\ H{\left(j\omega \right)}_{21} & H{\left(j\omega \right)}_{22} & \ldots & H{\left(j\omega \right)}_{2s}\\ \vdots & \vdots & \ldots & \vdots \\ H{\left(j\omega \right)}_{k1} & H{\left(j\omega \right)}_{k2} & \ldots & H{\left(j\omega \right)}_{ks} \end{array}\right]$$

where k denotes the maximum number of OFDM subcarriers and s represents the total number of OFDM frames samples in a single experiment. The WCSI frequency response of a single OFDM frame can be expressed as in Equation (2):

(2) $$H{\left(j\omega \right)}_{Frame}=\left[H\left(j\omega_{1}\right),H\left(j\omega_{2}\right),\ldots H\left(j\omega_{k}\right)\right]$$

The WCSI frequency response of each subcarrier contains complex value data, so we expressed the amplitude information in Equation (3):

(3) $$\left|H\left(j\omega_{k}\right)\right|=\sqrt{H{\left(j\omega_{k}\right)}_{real}{}^{2}+H{\left(j\omega_{k}\right)}_{img}{}^{2}}$$

$\left|H\left(j\omega_{k}\right)\right|$ is the amplitude of the *k*th subcarrier; amplitude information of WCSI helps identify the different activities performed by the subjects.

### 4.4. Data Preprocessing

The extracted data from WCSI is in a raw form, and it requires data preprocessing to get accurate, significant, and efficient analysis. In the first step, data is cleaned by removing and replacing missing or bad WCSI data. In the second step, the smoothing process is performed for removing noise by using low-pass filtering. In the final step, the grouping method is used to find correlations between the WCSI data values.

### 4.5. Features Extraction

The feature extraction method is helpful for the transformation of WCSI data, which translates collected WCSI data into significant trends in data. In addition, it is used for reducing the computation complexity and time by reducing the dimensions [51,52]. Therefore, feature extraction plays a crucial role in WCSI classification approaches. Presently, the statistical characteristics approach has been used for feature extraction. Statistical features used for developing the classification model are given in Table 4. Where the mean value gives information about the stable component of the signal, the standard deviation gives information about the degree of dispersion between the signal sampling points, the variance gives information about the fluctuations from the mean, the root mean square (RMS) value is a measure of the amplitude of a WCSI data, the peak-to-peak value is used for WCSI data amplitude range, the kurtosis is used to measure of the tailedness in the WCSI data, the skewness is used to represent symmetry of the WCSI data, the peak factor is used to detect whether there is an impact in the WCSI data, Interquartile range is used to obtain statistical dispersion and is equal to the difference between 75th and 25th percentiles, waveform factor is used to obtain the ratio of the RMS value to the average value of WCSI data, FFT functionality is used to extract frequency component with maximum and minimum values, spectral probability, signal energy, and spectrum entropy are used for the extraction of frequency domain analysis.

### 4.6. Classification

This research uses three popular machine learning algorithms to differentiate falls from other physical activities, and evaluates their performance. The accuracy of the machine learning algorithm depends on the type of dataset. The machine learning algorithms are used to develop models that predict physical activities based on WCSI data in the existence of uncertainty. These adaptive algorithms classify fall activity separately from standing, walking, running, and bending patterns by exploiting trends in the WCSI data. When the learning machine is trained to more experimental WCSI data, the processing machine improves its identification performance. All the experiment data are converted into a heterogeneous matrix. WCSI response data is a column vector where each row is labeled with the corresponding activity. The cross-validation (CV) model assessment technique is used to evaluate the performance of machine learning algorithms in making predictions on new WCSI datasets that have not been trained. We partition the known WCSI dataset, using a subset to train the machine learning algorithm and the left-over data for testing. A random 10-fold CV is used for original WCSI samples. These samples are randomly partitioned into 10 equal- sized WCSI data subsamples. Ninety percent of the WCSI data is used for training, while 10% is used for testing to develop a machine learning model. The accuracy of a model is used as a diagnostic measure to reflect the validated model results. The information about the conducted experiments is given in Table 5.

## 5. Results and Discussion

**Figure 3:**
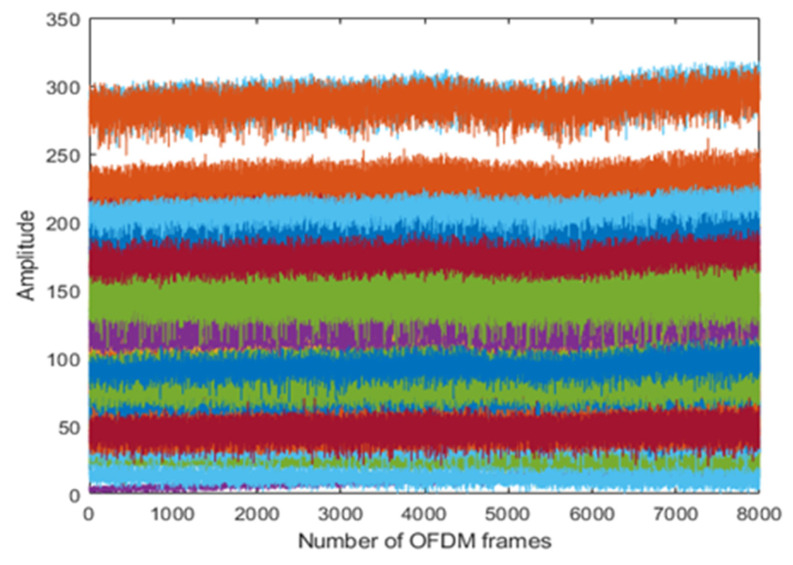
WCSI amplitude response of standing activity experiment.

**Figure 4:**
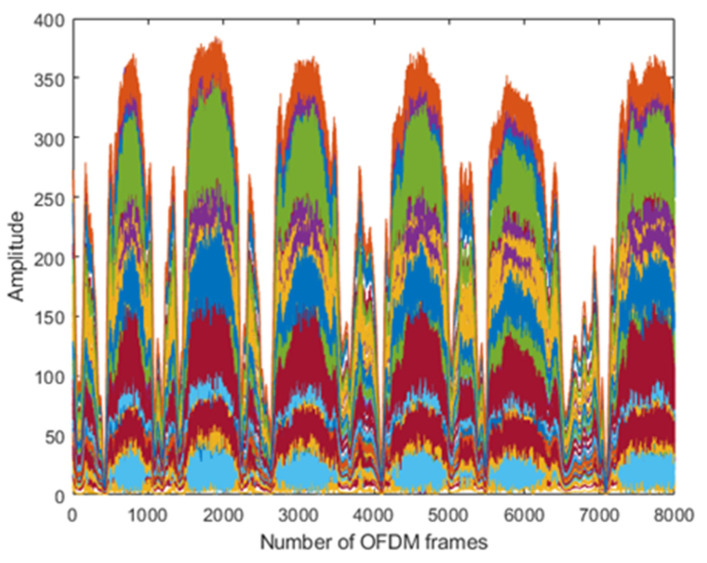
WCSI amplitude response of walking activity experiment.

**Figure 5:**
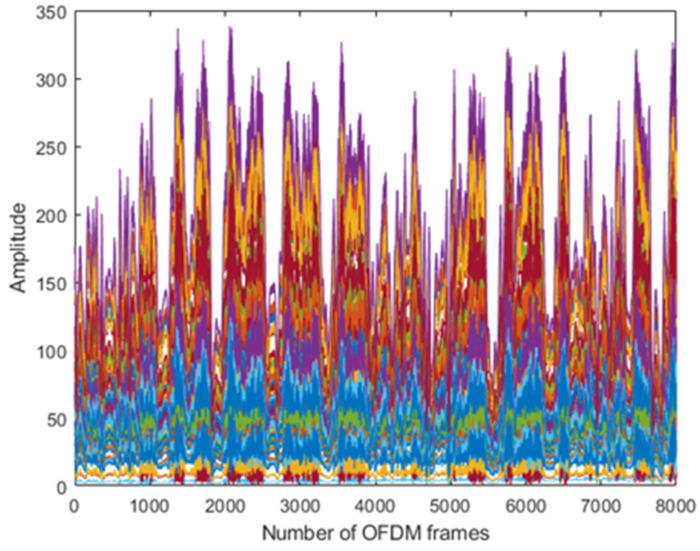
WCSI amplitude response of running activity experiment.

**Figure 6:**
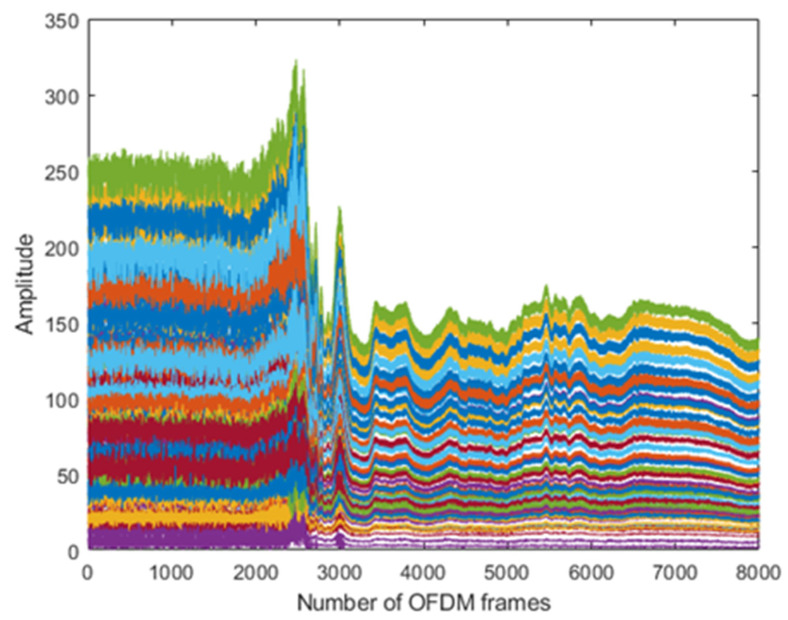
WCSI amplitude response of bending activity experiment.

**Figure 7:**
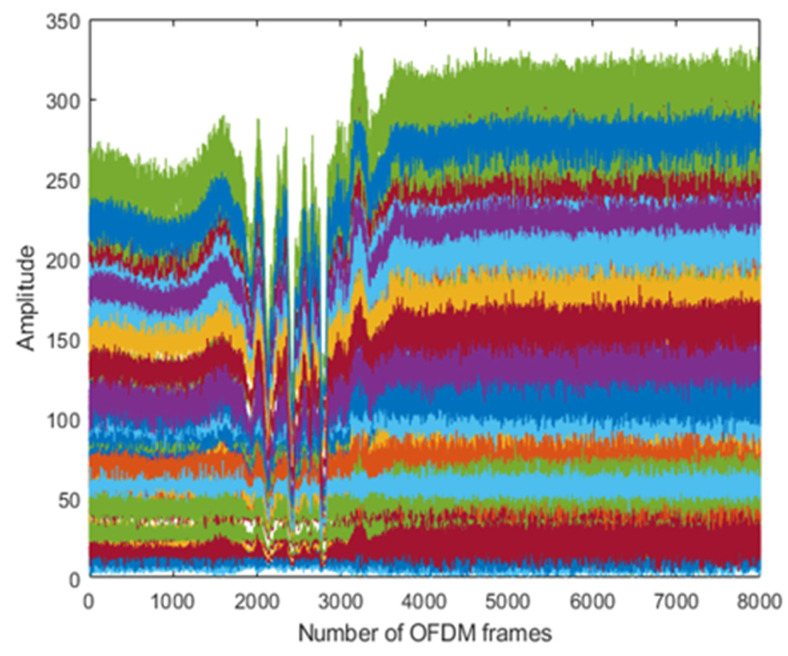
WCSI amplitude response of fall activity experiment.

The results are taken from the human physical activities experiments. The 64-subcarriers amplitude response of WCSI data is analyzed to detect physical activities. In Figure 3, the standing activity amplitude response of all the subcarriers in different colors is presented along the *y*-axis. The results show that WCSI amplitude response remains stable due to no human body movement in the standing activity over 8000 OFDM frames. In Figure 4, the walking activity amplitude response of all the subcarriers is presented in different colors along the *y*-axis. The results show that WCSI amplitude response varies slowly up and down due to slow human leg movement during the walking activity over 8000 OFDM frames. In Figure 5, the running activity amplitude response of all the subcarriers is presented in different colors along the *y*-axis. The results show that WCSI amplitude response varies rapidly up and down due to human leg movement during the running activity over 8000 OFDM frames. In Figure 6, the bending activity amplitude response of all the subcarriers is presented in different colors along the *y*-axis. The results show that WCSI amplitude response varies from top to bottom due to human upper body movement during the bending activity over 8000 OFDM frames. In Figure 7, the fall activity amplitude response of all the subcarriers is presented in different colors along the *y*-axis. The results show that WCSI amplitude response varies when the human body falls on the floor and then is stable during the fall activity over 8000 OFDM frames.

The confusion matrix presents the performance of the algorithm for each class. The confusion matrix determines the areas where the algorithm has performed well or poorly. The rows show the actual class, and the columns show the predicted class. The diagonal values give optimal results where the actual class and predicted class match. The observations from the actual class and predicted for physical activities are shown in Table 6. The results were achieved by applying machine learning algorithms on WCSI data collected from the SDR technology-based platform for monitoring falls. The performance of the different algorithms is shown in Table 7, which includes algorithm accuracy in percentage, observations per second (obs/s) for speed prediction, and time taken for training in seconds. The fine tree algorithm is best for WCSI data to classify physical activities, with an accuracy of 99.7%. Although fine KNN is less accurate, its prediction speed is higher than other algorithms, with more observations in unit time and less time in training the model on WCSI data.

## 6. Conclusions

In this research, smart sensing using SDR technology is exploited to detect falls during the quarantine period from other physical activities to reduce the chances of non-communicable as well as communicable diseases. USRP hardware is used to collect real-time data TTW of human physical activities that take place between the two antennas. The fine-grained WCSI data is extracted using OFDM technology to develop machine learning models. The machine learning model efficiently classifies fall activity separately from other physical activities. The performance of machine learning algorithms shows promising results, with the fine tree producing a high accuracy result of 99.7%, prediction speed of nearly 72,000 obs/s, and training in almost 9 s. This proof of concept can be further investigated to detect COVID-19 symptoms like shortness of breath, coughing, and cardiac arrest issues by exploring the smart sensing SDR technology platform.

Currently, the whole world is fighting against the novel coronavirus (COVID-19) and limiting their physical activities over time. In the future, smart sensing using SDR technology can cover a larger area by increasing the system gain, sampling rate, and Multiple Input Multiple Output (MIMO) antennas. Multiple subjects’ physical and sports activities can be recognized by extracting the signal reflection of each subject by examining the path of the reflected signals at multiple links. We can further reconstruct the signal profile of each subject as if only a single subject has performed an activity in the environment to facilitate multi-subjects’ activity recognition. The phase response is another solution to recognize multi-user activities in the same environments by measuring phase delays. The wireless channel is robust in nature, and it is hard to predict responses under changing surrounding environments. The time and frequency domain feature extraction of WCSI data can be exploited for better recognition accuracy by deploying state-of-the-art deep and machine learning algorithms.

## Figures and Tables

**Table 1:** Summary of non-contact human activity sensing literature.

Sr#	Technology	Activities Monitoring	Classification	Performance
1	Wi-Fi [23]	Moving human	SVM	99%
2	Wi-Fi [24]	Walking, running, sitting and falling	CSI-speed model	96%
3	Wi-Fi [25]	Human presence static and dynamic	Naïve Bayes	99%
4	Wi-Fi [26]	Walk, sit, stand, run	Deep auto-encoder	95%
5	Wi-Fi [27]	Human motion	HMM	94.2%
6	Wi-Fi [28]	Whole and partial-body movements	Machine learning	94.82%
7	Wi-Fi [29]	Upper, Lower and whole body	CNN	90%
8	Wi-Fi [30]	Bend, walk, sit down and squat	SVM	98.4%
9	Wi-Fi [31]	Walking, jogging and sitting	Deep auto-encoder	91.1%
10	Wi-Fi [32]	Standing and sitting	Soft-max regression	97.5%
11	Wi-Fi [33]	Walk, stand, empty and sit down	RNN	90%
12	Wi-Fi [34]	Moving area, path walking	Path matching	90.83%
13	Wi-Fi [35]	Quantifying running	SSF	93.18%
14	Wi-Fi [36]	Post-surgical fall	SVM	90%
15	Wi-Fi [37]	Breathing rate and falls	Machine learning	98%
16	Wi-Fi [38]	Danger Pose	SVM	96.23%
17	Wi-Fi [39]	Breathing and heart rate Patterns	DTW	94%
18	Wi-Fi [40]	Fall	SVM and RF	94%
19	Wi-Fi [41]	Fall	SVM	100%
20	Wi-Fi [42]	Respiration rate	EWMA	93.04%
21	Radar [43]	Sitting, standing, walking and jogging	K-mean	85%
22	Radar [44]	Walking, running, and crawling	KNN	93%
23	Radar [45]	Breathing	SVM	85%
24	Radar [46]	Standing, sitting, standing and fall	CNN	95.30%
25	SDR [47]	Standing, walking, crawling and lying	KNN	85%
26	SDR [48]	Standing up or sitting down	RF	96.70%
27	SDR [49]	Fractured ankle movement	CNN	98.98%
28	SDR [50]	Weight lifting	FKNN	99.6%

**Table 2:** Software-defined parameters setting of the non-contact smart sensing system.

Parameters	Values/Settings
Bits generation	128
Bits per symbol	2
Modulation type	QPSK
FFT size	64
Channel mapping Tx	1
Channel mapping Rx	2
Centre frequency Tx & Rx	2.45 GHz
Clock source & PPS source	Internal
Master clock rate Tx	200 MHz
Master clock rate Rx	200 MHz
Interpolation factor	250
Decimation factor	250
Enable burst mode	False
Transport data type Tx	int16
Transport data type Rx	int16
Output data type Tx	Same as transport data type
Output data type Rx	Same as transport data type
Serial number Tx	30AD2FE
Serial number Rx	30AD311
Gain Tx	80
Gain Rx	50
Samples per frames	80
Sampling rate	1000 samples/s

**Table 3:** Subject participation in experiments.

Sr. No	Subject	Age (Years)	Height (cm)	Weight (kg)	Body Structure
1	Male	25	168	55	Ectomorph
2	Male	27	180	95	Endomorph
3	Male	26	168	60	Mesomorph
4	Male	26	174	76	Mesomorph
5	Male	25	176	60	Ectomorph

**Table 4:** Statistical features expressions for classification.

Sr. No.	Features	Expression
1	Minimum	$Y_{min}=min\left(x_{i}\right)$
2	Maximum	$Y_{max}=max\left(x_{i}\right)$
3	Mean	$Y_{m}=\frac{1}{N}\overset{N}{\underset{i=1}{\sum }}x_{i}$
4	Standard deviation	$Y_{SD}=\sqrt[{2}]{\frac{1}{N-1}\overset{N}{\underset{i=1}{\sum }}{\left(x_{i}-Y_{m}\right)}^{2}}$
5	Variance	$Y_{V}=\overset{n}{\underset{i=1}{\sum }}{\left(x_{i}-Y_{m}\right)}^{2}$
6	Root mean square	$Y_{RMS}=\sqrt[{2}]{\frac{1}{N}\overset{N}{\underset{i=1}{\sum }}x_{i}{}^{2}}$
7	Peak to peak value	$Y_{PPV}=Y_{max}-Y_{min}\left(i=1,2,\ldots ,N\right)$
8	Kurtosis	$Y_{K}=\frac{\frac{1}{N}\sum_{i=1}^{N}{\left(\left|\begin{array}{c} x_{i} \end{array}\right|-Y_{PPV}\right)}^{4}}{Y_{RMS}{}^{4}}$
9	Skewness	$Y_{S}=\frac{\frac{1}{N}\sum_{i=1}^{N}{\left(\left|\begin{array}{c} x_{i} \end{array}\right|-Y_{PPV}\right)}^{3}}{Y_{RMS}{}^{3}}$
10	Peak factor	$Y_{P}=\frac{\max\left(x_{i}\right)}{Y_{RMS}}\;\left(i=1,2,\ldots ,N\right)$
11	Interquartile range	$Y_{IQ}=Q_{3}-Q_{1}$
12	Waveform factor	$Y_{WF}=\frac{N*Y_{RMS}}{\sum_{i=1}^{N}\left|\begin{array}{c} x_{i} \end{array}\right|}\;\left(i=1,2,\ldots ,N\right)$
13	FFT	$Y_{FFT}=\overset{N}{\underset{n=-N}{\sum }}x\left(n\right)e^{-j\frac{2\pi }{N}nd}$
14	Frequency Min	$Y_{fmin}=Min\left(Y_{FFT}\right)$
15	Frequency Max	$Y_{fmax}=Max\left(Y_{FFT}\right)$
16	Spectral Probability	$Y_{SP}=\frac{FFT{\left(d\right)}^{2}}{\sum_{i=-N}^{N}FFT{\left(i\right)}^{2}}$
17	Signal Energy	$Y_{SE}=\overset{N}{\underset{n=-N}{\sum }}{\left|p\left(d\right)\right|}^{2}$
18	Spectrum Entropy	$Y_{H}=\overset{N}{\underset{i=-N}{\sum }}p\left(d\right)\ln\left(p\left(d\right)\right)$

**Table 5:** Conducted experiments information.

Conducted Experiments Info	Quantity
Subjects participated	5
Activities performed	5
Experiment repetetion	10
Each experiment time	10 s
Total experiments	250
USRP devices	2
Computers	2
Antennas	2
Subcarriers	64
Classification algorithms	3
Total observations	16,000
Data size	2 MB
Predictors	18
Response classes	5
Validation method	10-fold CV

**Table 6:** Confusion matrix of machine learning algorithms on physical activities data.

Algorithms	Actual/Predicted	Standing	Walking	Running	Bending	Fall
**Fine KNN**	**Standing**	3200	0	0	0	0
	**Walking**	0	3200	0	0	0
	**Running**	0	5	3195	0	0
	**Bending**	0	0	10	3190	0
	**Fall**	0	0	0	0	3200
**Linear SVM**	**Standing**	3000	28	11	79	82
	**Walking**	0	3150	10	0	40
	**Running**	0	0	3104	20	76
	**Bending**	43	10	68	2994	85
	**Fall**	52	79	65	30	2974
**Fine Tree**	**Standing**	3200	0	0	0	0
	**Walking**	0	3185	15	0	0
	**Running**	0	2	3197	0	1
	**Bending**	0	3	8	3189	0
	**Fall**	0	0	18	0	3182

**Table 7:** Performance analysis of algorithms on physical activities data.

Algorithms	Accuracy (%)	Prediction Speed (obs/s)	Training Time (s)
Fine KNN	99.9%	~1400	415.48
Linear SVM	95.1%	~41,000	116.86
Fine Tree	99.7%	~72,000	9.074

## Abbreviations

ADC	Analog to Digital Converter
AI	Artificial Intelligence
AP	Access Point
BMS	Building Management Systems
CFR	Channel Frequency Response
CNN	Convolutional Neural Networks
CP	Cyclic Prefix
DAC	Digital to Analog Converter
DC	Direct Current
DTW	Dynamic Time Warping
DUC	Digital Up-Conversion
EWMA	Exponentially Weighted Moving Average
FDTW	Fast Dynamic Time Warping
FFT	Fast Fourier Transform
FRTCS	Fast and Robust Target Component Separation
HMM	Hidden Markov Model
IFFT	Inverse Fast Fourier Transform
ISI	Intersymbol Interference
KNN	K-Nearest Neighbors
LPF	Low Pass Filter
LSTM	Long Short-Term Memory
MIMO	Multiple Input Multiple Output
NIC	Network Interface Card
OFDM	Orthogonal Frequency Division Multiplexing
PADS	Passive Dynamic Velocity Moving People Detection System
QPSK	Quadrature Phase Shift Keying
RFA	Random Forest Algorithm
RNN	Recurrent Neural Network
SAR	Sports Automatic Recognition
SDR	Software Defined Radio
SSF	Stable Signal Fusion
SVM	Support Vector Machine
TTW	Through the Walls
WCSI	Wireless Channel State Information
Wi-OAR	Wireless Occupant Activity Recognition System
WSN	Wireless Sensor Network
